# Feedback about action performed can alter the sense of self-agency

**DOI:** 10.3389/fpsyg.2014.00145

**Published:** 2014-02-25

**Authors:** Neeraj Kumar, Jaison A. Manjaly, Krishna P. Miyapuram

**Affiliations:** Cognitive Science Program, Indian Institute of Technology GandhinagarAhmedabad, India

**Keywords:** sense of agency, motor control, forward model, error monitoring mechanisms, Flanker task, error feedback, action intention

## Abstract

Sense of agency refers to the sense of authorship of an action and its outcome. Sense of agency is often explained through computational models of motor control (e.g., the comparator model). Previous studies using the comparator model have manipulated action-outcome contingency to understand its effect on the sense of agency. More recent studies have shown that cues related to outcome, priming outcome and priming action have an effect on agency attribution. However, relatively few studies have focused on the effect of recalibrating internal predictions on the sense of agency. This study aims to investigate how feedback about action can recalibrate prediction and modulates the sense of agency. While participants performed a Flanker task, we manipulated the feedback about the validity of the action performed, independent of their responses. When true feedback is given, the sense of agency would reflect congruency between the sensory outcome and the action performed. The results show an opposite effect on the sense of agency when false feedback was given. We propose that feedback about action performed can recalibrate the prediction of sensory outcome and thus alter the sense of agency.

## INTRODUCTION

Our ability to interact with the environment through action is an essential aspect of our day-to day-life. Intention to act, preparation to move, generating motor commands, and sensory feedback are some of the underlying aspects of sensorimotor experience and sense of agency (Haggard et al., 2002). Sense of agency is the experience of authorship of an action (Gallagher, 2000). Multiple theories have been proposed to describe mechanisms responsible for the sense of agency (see David et al., 2008 for a review). Previous studies have manipulated action-outcome contingency to understand its effect on the sense of agency. More recent studies have shown that cues related to outcome, priming outcome, and priming action have an effect on agency attribution (Aarts et al., 2005, 2009; Aarts, 2007, Dijksterhuis et al., 2008; van der Weiden et al., 2010; Wenke et al., 2010). However, relatively few studies have focused on the effect of modulating action *per se* on self-action perception (Synofzik et al., 2006). The current study investigates whether feedback about the action performed could modulate the sense of agency. We hypothesize that manipulating feedback would alter agency attribution by recalibrating the prediction of sensory outcome.

The sense of agency for intended action has been successfully explained by the comparator model (Frith, 1992), which consists of the inverse and forward models. The inverse model identifies motor commands to achieve a desired goal state and the forward model predicts sensory consequences of motor actions. These models are represented within the motor-control system (Wolpert et al., 1998). The forward model is principally responsible for sense of agency because it generates an efference copy of motor commands of intended action (Frith, 1992) and predicts the corresponding sensory consequences. The predicted sensory consequence is matched against the subsequent actual sensory information (i.e., outcome). Sense of agency will be experienced for those events for which the predicted and the sensed information match. In case of a mismatch, the sense of agency will be absent or could be attributed to an external agent.

In case of unintended actions (such as errors), there would be a mismatch between the predicted outcome of the intended action and the sensory outcome generated by the action performed. In this case, the comparator model would infer an absence of self-agency. However, Sato and Yasuda (2005) showed that sense of agency depends on the congruency of an action and its outcome not only when the action was intended, but also when it was unintended.

One of the explanations for sense of agency in case of unintended actions comes from studies on error monitoring mechanisms (van Schie et al., 2004; Yordanova et al., 2004; Knoblich and Sebanz, 2005). These studies suggest that error-monitoring signal detects conflict between several possible actions, which in turn can be used to readjust the prediction of the sensory outcome (i.e., conflict between the action performed and the sensory outcome). Knoblich and Sebanz (2005) suggested that, this readjustment could either serve as a direct indication of agency or it could influence post-hoc judgment of agency. These suggestions, although theoretically important, have not been formally tested. Therefore, in the present study, we attempt to test how feedback about the validity of action would modulate the sense of agency.

In this study, participants performed a Flanker task (Eriksen and Eriksen, 1974) and generated a tone outcome similar to Sato and Yasuda (2005). The tone generated was either congruent or incongruent with the action performed, an association that was learnt during a training phase (see Materials and Methods). Before the occurrence of the outcome, feedback about the validity of the action performed was provided. That is, the feedback could either be true or false, unbeknownst to the participants. When true feedback is given, participants should have higher sense of agency for congruent tones, but low sense of agency for incongruent tones. We hypothesize that a false feedback would reverse the judgment about sense of agency (**Table 1**). Thus, when a false feedback is given, participants might perceive a discrepancy between performed and intended action. This would lead to a readjustment of the prediction of sensory outcome (tone) based on an alternate action, instead of the actual action performed. Therefore, validity of feedback about the action performed would determine upcoming sensory consequences and the agent would attribute agency accordingly.

**Table 1:** Hypothesized sense of agency as a function of feedback validity and tone congruency with the action performed.

	Congruent tone	Incongruent tone
True feedback	High self-agency	Low self-agency
False feedback	Low self-agency	High self-agency

## MATERIALS AND METHODS

### PARTICIPANTS

Fifteen undergraduate students (Mean age = 19.4 years, 13 males and two females, range: 19–28 years) participated in the study. We repeated the experiment with 15 participants (Mean age = 22.5 years, 10 male and five female, range: 20–26 years). All the participants were right-handed with normal or corrected to normal vision. They gave informed consent and were paid for the participation.

### STIMULI

**FIGURE 1:**
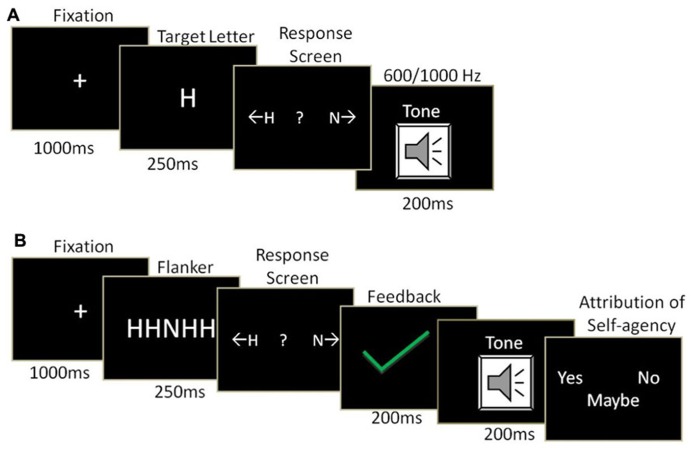
**Trial structure of (A) training phase and (B) test phase.** In training phase, participants learned the association between action (key press towards target letter) and outcome (tone). In test phase, target letter was flanked by either congruent or incongruent letters. We manipulated the feedback about the validity of action performed, and tone congruency with intended action. The participants reported self-agency ratings in the form of “Yes”, “No”, or “Maybe.”

Stimuli consist of two target letters “H” & “N.” In an initial training phase, the target letters were assigned two corresponding key presses (left/right arrow key). The key presses were, in turn, associated with two tones of 600 and 1000 Hz as sensory outcomes, (**Figure 1A**). In the test phase (**Figure 1B**), we used the Eriksen Flanker task (Eriksen and Eriksen, 1974) in which the target letter was flanked by two distracter letters on each side. The flankers could be either congruent (e.g., HHHHH) or incongruent (e.g., NNHNN) with the target letter. The sensory outcomes in the test phase (i.e., Flanker task) were of the same tones as those of the training phase. An action of pressing a button (left/right arrow key) was required for each target presentation, prior to the sensory outcome. Feedback was introduced for the action made in response to the target letter in the Flanker task prior to the sensory outcome (i.e., tone). A green tick mark (✓) or a red cross mark (χ) was used to indicate feedback, which was manipulated independently from the actual response (see Procedure). The assignment of target stimuli to action buttons, and to the tone outcome was consistent throughout the experiment (i.e., training and test phase) for each participant and counterbalanced across participants. The tones were identical in duration and sound pressure throughout the experiment.

### PROCEDURE

The participants entered a dimly lit room and were seated in front of a computer screen with a pair of headphones (Sony MDR-ZX700, Over-the-ear). They were provided a printed instruction sheet that explained the task procedure. To induce the setting of the study pertaining to sense of agency, the instruction sheet mentioned that another participant sitting in an adjacent room could also generate the outcome. The experiment consisted of a training phase and a test phase. In the training phase, the target letters (H or N) did not have any flankers. The Participants learnt the association between a key press (right or left arrow key, in response to target letters) and a corresponding sensory outcome (600 or 1000 Hz tone). In the test phase, participants responded to the same target letters in a Flanker task. A Feedback was introduced whether response was correct or not, followed by sensory outcome (600 Hz or 1000 Hz tone). A Self-report measure of the sense of agency pertaining to the tone outcome was obtained. We manipulated two within-subject factors – (a) type of sensory outcome (congruent tone or incongruent tone) with the key press, and (b) validity of feedback (true feedback or false feedback) for key press. Participants completed a brief practice session (30 trials) before the training and the test phases, to familiarize themselves with the task procedures.

In the training phase (See **Figure 1A**), the participants performed 300 trials. On each trial, 1000 ms after fixation onset, the target stimulus (i.e., “H” or “N”) was presented for 250 ms on the center of the screen. The participants were required to press left or right arrow keys, assigned to target letters ‘H’ and ‘N’, respectively. The responses were made using the index and the ring fingers of the dominant hand. To reduce any possible memory effects, key assignment was displayed on the screen. The participants were instructed to press the assigned key upon the appearance of a target letter. Further, the action performed (key press) would evoke a certain tone (600 Hz tone or a 1000 Hz). The tone was presented immediately after the key press for 200 ms. Incorrect trials resulted in same tone outcome that was contingent on a key press and were excluded from data analysis.

In the test phase (See **Figure 1B**) the participants performed a Flanker task for 200 trials. Each trial started with the onset of a centrally presented fixation sign. After 1000 ms, a five-letter array (i.e., HHHHH, NNNNN, HHNHH, or NNHNN) was presented for 250 ms. Participants were instructed to respond to one of the two target letters (central H or N) with their right index or ring fingers, respectively. An immediate feedback was provided for 200 ms, which could be a true (green tick for correct response or red cross for wrong response, 60% of total trials) or a false feedback (red cross for a correct response or green tick for a wrong response, 40% of total trials). After 200 ms of offset of feedback, a tone was presented through the headphones for 200 ms either congruent or incongruent with the intended key-press. The participants were then asked to rate their sense of self-agency (“I was the one who produced the tone”). The responses could be “Yes”, “No,” or “Maybe”. The left, right, and down arrow keys on the keyboard were used to record these responses using index, ring, and middle fingers of the right hand, respectively. To prevent demand effects and any other possible bias in responses, such as motor preparation, the key assigned for “Yes” and “No” was randomized between the left and right arrow keys across trials. The “Down” arrow key was consistently assigned for “Maybe.” Our measure of sense-of-agency was based on self-report. To minimize the influence of experimental demand, an additional question on sense of ownership was included. While the sense of agency would be modulated by experimental conditions, the sense of ownership would remain high in all experimental conditions, serving as a control measure (Sato and Yasuda, 2005). After every 10 trials, participants rated the sense of agency (“I was the one who produced the tone”) and the sense of ownership (“I was the one who was listening to the tone”) by moving a slider bar with a mouse on a continuous scale of 0–100 (see **Figure 1B**). The Presentation of both the questions was counterbalanced across the trials. The experiment was designed and presented using Psychophysics toolbox (Brainard, 1997) in MATLAB (Mathworks Inc.).

In this experiment (hereafter Experiment 1) we assumed that participants believed in the feedback. We did not do an explicit debriefing after the experiment to avoid any information exchange among the participants. To verify our assumption, we repeated the experiment (hereafter Experiment 2) with a debriefing session at the end. The Participants were asked to report the percentage of correct responses they had made out of total trials. They were also asked to rate their belief in the accuracy of feedback on a scale of 0 (Disagree) to 10 (Agree).

### DATA ANALYSIS

In the training phase, we measured the reaction time and accuracy of responses. In the test phase, we measured the reaction time and accuracy towards the central target letter of the Flanker stimuli. For each trial, we recorded self-report ratings of the sense of agency (“Yes”, “No,” or “Maybe”) that were transformed into discrete numerical values of 1, 0, and 0.5, respectively. We also recorded response times for the self-report ratings. The sense of agency and the sense of ownership were measured on a continuous scale of 0–100 after every 10 trials.

Reaction time to a target letter in the training and test phase (Flanker task) was analyzed separately using two-sample *t*-tests. The rating of sense of agency was analyzed using repeated measure analysis of variance (ANOVA) with three factors: Tone congruency with key press (two levels – congruent and incongruent tone) × Validity of feedback (two levels – true and false feedback) × Flanker type (congruent and incongruent with target letter). The sense of ownership rating and the reaction time for the rating of sense of agency were analyzed in a similar repeated measure ANOVA. The sense of agency rating, reaction time to target letter, and sense of ownership were analyzed only for the correct response trials (95.53% in Experiment 1 and 94.82% in Experiment 2).

## RESULTS

Between Experiments 1 and 2, only the latter had a debriefing session. In Experiment 2, two participants were removed from further analysis because they reported more than 95% correct trials and rated low belief (Mean = 2.5, on a scale of 0–10) on feedback. The remaining 13 participants reported an average of 65.92% (SD = 5.58) correct trials and they rated high belief (Mean = 7.92, SD = 0.86) in feedback. Comparable estimates of correct trials (65%) with the percentage of true feedback (60%) suggest that the participants believed in feedback (χ^2^ = 1.04, *p *= 0.30).

### SENSE OF AGENCY

**FIGURE 2:**
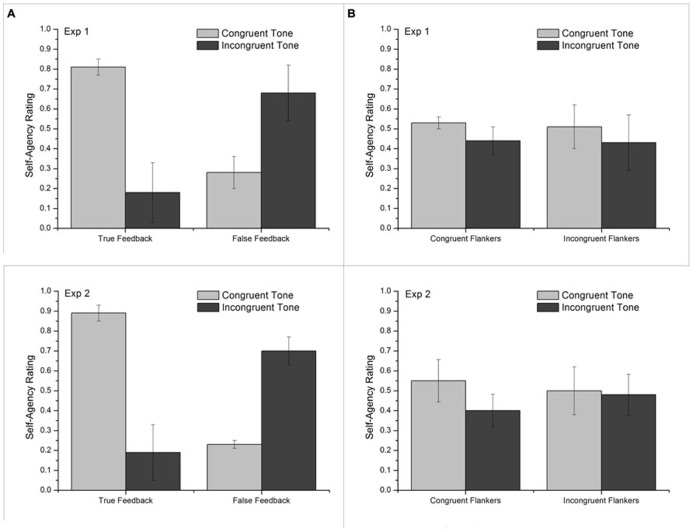
**Sense of self-agency calculated from discrete scale (e.g., Yes, No, or Maybe) in Experiment 1 (top panels) and Experiment 2 (bottom panels).** Standard deviations are plotted as error bars. **(A)** Average self-agency rating in true and false feedback conditions. **(B)** Average self-agency rating in congruent and incongruent flankers.

Repeated measures ANOVA on the sense of agency rating (discrete values) revealed main effect of tone congruency, whereas no significant main effect of feedback and flanker was found. However, more crucially we found significant interaction between tone congruency and validity of feedback. This suggests that effect of congruency of tone on sense of self-agency was dependent on feedback. There was no significant interaction between flanker and feedback, and flanker and tone congruency (See **Table 2**). Further paired-sample *t*-test revealed that the sense of self-agency was significantly reduced for congruent tone and increased for incongruent tone when feedback given was false (See **Table 3**; **Figure 2**).

**Table 2:** Repeated measure ANOVA on self-agency rating, response time for agency rating, and ownership rating of Experiments 1 and 2.

Measures	Source	Experiment 1	Experiment 2
Self-agency rating	Feedback	*F*(1,14) = 0.68, *p *= 0.42	*F*(1,12) = 1.54, *p *= 0.23
	Flanker congruency	*F*(1,14) = 1.22, *p *= 0.28	*F*(1,12) = 1.22, *p *= 0.29
	Tone congruency	*F*(1,14) = 9.56, *p *< 0.01	*F*(1,12) = 5.75, *p *= 0.03
	Tone congruency × feedback	*F*(1,14) = 852.01, *p *< 0.01	*F*(1,12) = 758.64, *p *< 0.01
	Flanker × feedback	*F*(1,14) = 0.24, *p *= 0.63	*F*(1,12) = 0.72, *p *= 0.41
	Flanker × tone congruency	*F*(1,14) = 0.09, *p *= 0.76	*F*(1,14) = 0.008, *p *= 0.93
Agency rating time	Feedback	*F*(1,14) = 3.12, *p *= 0.09	*F*(1,12) = 3.72, *p *= 0.07
	Flanker congruency	*F*(1,14) = 0.07, *p *= 0.79	*F*(1,12) = 1.58, *p *= 0.49
	Tone congruency	*F*(1,14) = 2.12, *p *= 0.16	*F*(1,12) = 0.515, *p *= 0.48
	Tone congruency × feedback	*F*(1,14) = 11.04, *p *< 0.01	*F*(1,12) = 6.61, *p *= 0.02
Ownership rating	Feedback	*F*(1,14) = 0.02, *p *= 0.88	*F*(1,12) = 0.08, *p *= 0.78
	Tone congruency	*F*(1,14) = 0.54, *p *= 0.47	*F*(1,12) = 0.67, *p *= 0.42
	Tone congruency × feedback	*F*(1,14) = 1.51, *p *= 0.23	*F*(1,12) = 1.32, *p *= 0.28

**Table 3:** Mean ± SD self-agency rating in different conditions of Experiment 1 and 2.

Type of tone		False feedback	True feedback	Paired-sample *t*-test
Congruent tone	Experiment 1	0.28 ± 0.08	0.81 ± 0.04	*t*(14) = 22.13, *p *< 0.01
	Experiment 2	0.23 ± 0.02	0.89 ± 0.04	*t*(12) = 25.14, *p *< 0.01
	Experiment 2 (excluded participants)	0.75	0.79	
Incongruent tone	Experiment 1	0.68 ± 0.10	0.18 ± 0.15	*t*(14) = 10.67, *p *< 0.01
	Experiment 2	0.70 ± 0.07	0.19 ± 0.14	*t*(12) = 12.80, *p *< 0.01
	Experiment 2 (excluded participants)	0.65	0.17

**FIGURE 3:**
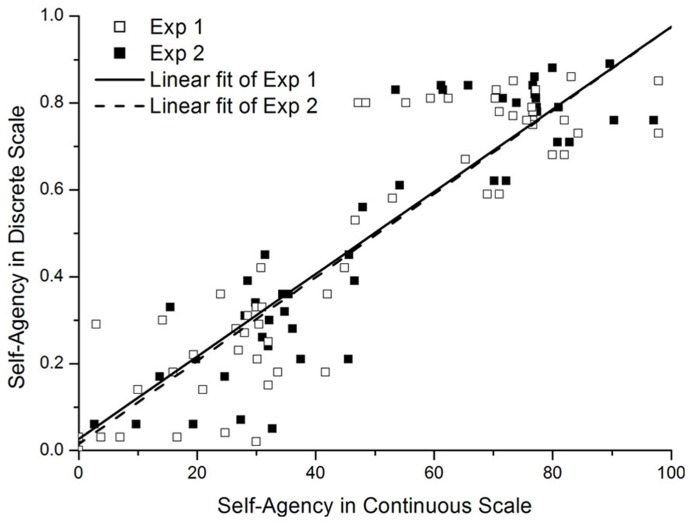
**Correlation between the self-agency ratings obtained from continuous and discrete scale**.

To explore any difference in the self-agency rating due to two different rating scales, we performed bivariate correlation between ratings from discrete (Yes, No, or Maybe) and continuous scales (0–100). A significant correlation between these two measures was obtained [Experiment 1: *r*(60) = 0.89, *p *< 0.01, Experiment 2: *r*(52) = 0.90, *p* < 0.01 ] (See **Figure 3**).

### RESPONSE TIME ON AGENCY RATING

The response time taken to rate the sense of self-agency was analyzed using repeated measures ANOVA with three factors – type of Flanker (2), type of feedback (2), and tone congruency with key press (2). This analysis revealed significant interaction between feedback and tone, but no main effect of tone congruency or feedback was found. Moreover, flanker congruency with target letter had no main effect on response time to attribute self-agency (See **Table 2**). Further, pair-wise comparison showed that response times for agency rating in congruent tone conditions were significantly higher [Experiment 1: *t*(14) = 3.14, *p *< 0.01; Experiment 2: *t*(12) = 2.85, *p *< 0.01] in false feedback (Experiment 1: 1.91 ± 0.58 s, Experiment 2: 1.80 ± 0.41 s) than in true feedback conditions (Experiment 1: 1.42 ± 0.12 s, Experiment 2: 1.57 ± 0.19 s). In incongruent tone conditions, response times for rating sense of agency were significantly lower [Experiment 1: *t*(14) = 2.93, *p *< 0.05; Experiment 2: *t*(12) = 2.85, *p *< 0.01] in false feedback (Experiment 1: 1.47 ± 0.11 s, Experiment 2: 1.40 ± 0.14 s) as compared to true feedback conditions (Experiment 1: 1.65 ± 0.12 s, Experiment 2: 1.72 ± 0.15 s). The Participants took similar amounts of time [Experiment 1: *t*(14) = 1.41, *p *= 0.17; Experiment 2: *t*(12) = 1.11, *p *= 0.28] to attribute agency when they received false feedback and incongruent outcome as compared to true feedback and congruent outcome.

### REACTION TIME TO TARGET LETTER

To check the manipulation effect of the Flanker task, we analyzed the reaction time to target letter for correct and incorrect trials in both training and test phases, separately. In the training phase the participants were significantly faster [Experiment 1: *t*(14) = 2.97, *p *= 0.01; Experiment 2: *t*(12) = 2.75, *p *< 0.01] in incorrect responses (Experiment 1: 0.32 ± 0.15 s, Experiment 2: 0.35 ± 0.17 s) than correct responses (Experiment 1: 0.53 ± 0.18 s, Experiment 2: 0.55 ± 0.15 s). The participants responded correctly in 98.95 and 97.84% of total trials (300) in the training phase of Experiments 1 and 2, respectively. In the test phase, participants were significantly slower [Experiment 1: *t*(14) = 3.34, *p *< 0.01; Experiment 2: *t*(12) = 5.82, *p *< 0.01] when they made incorrect responses (Experiment 1: 1.3 ± 0.5 s, Experiment 2: 1.23 ± 0.28 s) in comparison to correct responses (Experiment 1: 0.75 ± 0.06 s, Experiment 2: 0.78 ± 0.04 s). The participants responded correctly in 95.53 and 94.82% of total trials (200) in the test phase of experiments 1 and 2, respectively. The participants were significantly slower [Experiment 1: *t*(14) = 6.87, *p *< 0.01; Experiment 2: *t*(12) = 13.16, *p* < 0.01] in responding to the target letter when its flankers were incongruent (Experiment 1: 0.83 ± 0.07 s, Experiment 2: 0.86 ± 0.06 s) as compared to congruent (Experiment 1: 0.67 ± 0.08 s, Experiment 2: 0.63 ± 0.03 s).

### SENSE OF OWNERSHIP RATING

Rating for sense of ownership was analyzed through repeated measure ANOVA with two within subject factors (tone congruency with prediction, and feedback). There was no main effect of either tone congruency with prediction or feedback on sense of ownership. No significant effect of interaction between tone congruency and feedback on sense of ownership was found (See **Table 2**).

## DISCUSSION

The present study aims to investigate how sense of agency is modulated by feedback about the validity of performed action. Previous research has investigated how factors such as priming the action, priming the outcome, or varying the characteristics of the outcome affect the sense of agency. However, in this study, we address how feedback about action validity affects sense of agency. The results indicate that when true feedback was given, an increased higher sense of agency was observed for congruent as compared to incongruent outcome for the performed action. The novelty of this study lies in the finding that participants felt a higher sense of agency for the incongruent outcome when false feedback was given. This result suggests that the participants readjusted the prediction of outcome based on the feedback given, and attributed the sense of agency accordingly.

In this study, the participants learned to predict a specific outcome (a pair of tones) contingent on an action performed (a preceding key press). Previous research has shown that manipulating outcome (e.g., tone congruency with the prediction) alters the sense of agency (Fourneret and Jeannerod, 1998; Sato and Yasuda, 2005). Our results confirm the effect of outcome manipulation with a significant main effect for tone congruency on the sense of agency. This is consistent with the comparator model as the prediction generated by the intended action (congruent tone) does not match with the outcome (incongruent tone), i.e., when true feedback was given (consistent with actual action performed), an increased sense of agency was reported for congruent as compared to incongruent tones.

Sato and Yasuda (2005) have found that sense of agency is experienced for both intended and unintended actions (i.e., erroneous actions). In their study, Flanker stimuli introduce ambiguity over action performed and created room for unintended actions /errors. When an error is made, intended action and actual action were different. Hence, predictions of sensory outcomes from intended and actual actions are not the same. Sense of agency was found to be higher when the outcome matched with prediction based on actual action. In contrast, the sense of agency was low when outcome matched with prediction based on intended action, but did not match with actual action (Sato and Yasuda, 2005). These results suggest that a readjustment of prediction of sensory outcome has occurred through error monitoring mechanisms (van Schie et al., 2004; Yordanova et al., 2004).

We used Flanker stimuli similar to Sato and Yasuda (2005) with external feedback about validity of the action performed. The feedback acts as an intermediate outcome before the final tone. If sense of agency depends on intended action independent of feedback, then we should observe a high sense of agency for outcomes (i.e., tone) that are congruent with prediction based on intention (i.e., target letter). On the other hand, if feedback modulates sense of agency, then higher sense of agency would be experienced for incongruent outcome with intended actions when a false feedback is given. Our results support the latter hypothesis that sense of agency had a strong interaction between feedback and outcome congruency with intended action. As expected, sense of agency was high for congruent and low for incongruent outcomes when true feedback was given. In contrast, participants attributed high self-agency for incongruent outcome and low self-agency for congruent outcome, when false feedback was given. We speculate that in case of false feedback, the altered agency attribution would reflect recalibration of sensory predictions.

We assume that participants would believe that experimentally given feedback was always true. We have replicated findings from Experiment 1 with a subset of participants in Experiment 2 who believed in feedback. The estimated correct number of trials matched close to actual number of true feedback trials in Experiment 2. However, during the debriefing session, two of our participants expressed their doubts on the validity of feedback. In the absence of any modulation of the sense of agency by feedback, self-agency should be reported to be high for congruent tones and low for incongruent tones irrespective of feedback. However, for incongruent tones, these participants rated high self-agency with false feedback (Mean = 0.65) in comparison to true feedback (Mean = 0.17; see **Table 2**). This confirms our findings partially, even when these participants explicitly mentioned low belief in the feedback given. Since there are only two such reports, we have not discussed them in detail.

There could be four possibilities for the modulation of the sense of agency based on the feedback regarding validity of action. (1) Participants could infer that perception of target letter was wrong, (2) Participants could modulate inference of sense of self-agency after the outcome, (3) Participants could readjust the notion of executed motor program, i.e., they inferred that intended action was not actual action, but believed that the other possible action was performed, and (4) Participants could readjust the prediction of sensory outcome from intended action to the outcome of other possible action, irrespective of actual action.

The first possibility is ruled out because we did not find a main effect of flanker on sense of agency. We also argue against any misidentification of key to target map because this information was explicitly displayed until the response was made. Further, these mappings did not change across trials for a given participant. We also rule out the second possibility as low sense of agency was reported for congruent tones with false feedback. If the reconstruction happens after the outcome, participants should have reported a higher sense of agency for congruent tones irrespective of whether the feedback was true or false.

Our results support the third and the fourth possibilities and show that there is a recalibration of sensory prediction based on feedback. However, it is not possible to dissociate whether the recalibration is based on motor program level (i.e., possibility 3 above) or at the level of predicted outcome (i.e., possibility 4 above). According to this scheme, feedback can override predictions by intended action. Hence, predictions by efference copy of the comparator model would pertain to recalibrated motor program, rather than intended actions alone. Our results support the previous findings of Sato and Yasuda (2005) regarding error trials. In case of error, an internal feedback might be generated, which would in turn recalibrate the predictions of sensory outcome. One further support for our argument comes from the analysis of response times for attribution of sense of agency. Results showed that participants take similar amounts of time to attribute agency when they received false feedback and incongruent outcome as compared to true feedback and congruent outcome. This shows that participants were already expecting the incongruent outcome based on false feedback. While sense of agency was modulated with feedback, such an effect was not apparent on the sense of ownership. Intact sense of ownership in case of false feedback is similar to previous studies on prediction and agency attribution (Sato and Yasuda, 2005). It provides support to previous claims that sense of ownership is driven by mere presence of sensory consequences and is not affected by the characteristics of sensory prediction (Sato and Yasuda, 2005).

Future studies investigating the role of feedback in sense of agency could focus on dissociating recalibration at motor program level vs. predicted outcome level. The feedback stimulus in this study is assumed to be associated with the action performed as part of the Flanker task. However, feedback precedes tone outcome associated with key press. It would be necessary to verify further the exact role of the feedback cue on agency attribution. It also remains to be investigated whether the sense of agency would be modulated or not, if feedback cues were replaced by a prime for outcome. Support for our argument for the role of feedback in recalibration of prediction can be obtained by EEG studies focusing on error related negativity. Alternatively, such a study could also clarify whether the modulation in sense of agency is simply a *post hoc* reconstruction based on observed outcome with no prediction involved.

In summary, our hypothesis and results suggest that feedback about the validity of action can recalibrate sensory predictions and alter agency attribution. Hence, studies pertaining to sense of self-agency should also include the effect of feedback (which could also be internally driven) beyond the action – outcome contingency driven attribution.

## Conflict of Interest Statement

The authors declare that the research was conducted in the absence of any commercial or financial relationships that could be construed as a potential conflict of interest.
